# Molecular chaperones are nanomachines that catalytically unfold misfolded and alternatively folded proteins

**DOI:** 10.1007/s00018-014-1627-y

**Published:** 2014-04-24

**Authors:** Rayees U. H. Mattoo, Pierre Goloubinoff

**Affiliations:** Department of Plant Molecular Biology, Faculty of Biology and Medicine, University of Lausanne, Biophore Building, 1015 Lausanne, Switzerland

**Keywords:** Hsp70, Hsp110, Hsp40, GroEL, Disaggregase, Polypeptide unfoldase, Holdase, Translocase

## Abstract

By virtue of their general ability to bind (hold) translocating or unfolding polypeptides otherwise doomed to aggregate, molecular chaperones are commonly dubbed “holdases”. Yet, chaperones also carry physiological functions that do not necessitate prevention of aggregation, such as altering the native states of proteins, as in the disassembly of SNARE complexes and clathrin coats. To carry such physiological functions, major members of the Hsp70, Hsp110, Hsp100, and Hsp60/CCT chaperone families act as catalytic unfolding enzymes or unfoldases that drive iterative cycles of protein binding, unfolding/pulling, and release. One unfoldase chaperone may thus successively convert many misfolded or alternatively folded polypeptide substrates into transiently unfolded intermediates, which, once released, can spontaneously refold into low-affinity native products. Whereas during stress, a large excess of non-catalytic chaperones in holding mode may optimally prevent protein aggregation, after the stress, catalytic disaggregases and unfoldases may act as nanomachines that use the energy of ATP hydrolysis to repair proteins with compromised conformations. Thus, holding and catalytic unfolding chaperones can act as primary cellular defenses against the formation of early misfolded and aggregated proteotoxic conformers in order to avert or retard the onset of degenerative protein conformational diseases.

## Introduction

Anfinsen demonstrated that under optimal non-physiological conditions of low protein concentrations and low temperatures, the primary amino acid sequence of a polypeptide contains the necessary instructions for its spontaneous acquisition of a narrow range of relatively stable but dynamic functional structures, generally referred to as the “native state” [1]. Yet, the refolding process is often inefficient because hydrophobic residues that in stress-unfolded or de novo-synthesized polypeptides become abnormally exposed to the aqueous phase, may spontaneously seek intra-molecular stability by forming wrong beta sheets and improper inter-molecular ensembles generally called aggregates. Because of cooperativity, a simple increase in the number of surface-exposed hydrophobic residues may result in a synergic increase of the affinity between several misfolded polypeptides. Thus, aggregate-entrapped polypeptides may become precluded from dissociating and reaching their native state within a biologically relevant time-scale [2, 3]. Moreover, aggregates may be cytotoxic, especially to animal cells, and cause aging-induced degenerative disorders, such as Parkinson’s, Huntington’s and Alzheimer’s diseases [4]. In youth, however, a cellular network composed of molecular chaperones and of chaperone-controlled proteases can efficiently counteract toxic protein aggregation by a mechanism generally termed as “holdase”, corresponding to the non-catalytic tight binding of aggregation-prone misfolding intermediates to the chaperone surface. For a general review on the main chaperone families, their structures, and their anti-aggregation activities, see [5]. Here, we focus on chaperones that seem to function as catalytic unfolding enzymes and are of importance in combating early proteotoxic intermediates in protein conformational diseases.

Several independent studies have reported unfolding of misfolded polypeptides by chaperonins and Hsp70 chaperones [6–11]. Recently, members of conserved chaperone families Hsp70, Hsp110, and Hsp60/CCT have been shown to drive catalytic polypeptide unfolding activity, where sub-stoichiometric quantities of chaperones could process a molar excess of high-affinity misfolded substrates into low-affinity native products [12, 13]. A clear understanding of the passive “holding” and the catalytic unfolding mechanisms by which some chaperones can oppose the formation of toxic protein conformers, and others actively revert already-formed toxic aggregated conformers into harmless native or degraded polypeptides, is central to the design of new therapeutic solutions to protein conformational diseases. Here, we review the different molecular functions of chaperones and critically discuss the adequacy of the terms that are used in the literature to describe them.

## The role of chaperones in protein misfolding diseases

Under physiological conditions, molecular chaperones and proteases control house-keeping processes of cellular proteostasis, such as assisting the proper de novo folding of polypeptides exiting the ribosome, or of cytoplasmic proteins exiting the import pores in the *endoplasmic reticulum* lumen or the mitochondrial matrix. Molecular chaperones also activate or inhibit various signaling pathways [14–16]. For example, Hsc70 regulates SNARE complexes [17, 18]. After exocytosis, when the* cis*-SNARE complex is stuck on the target membrane, the AAA+ ATPase *N*-ethylmaleimide sensitive factor disassembles it and after disassembly, Hsc70 together with cysteine-string protein-alpha and small guanine-rich tetratricopeptide protein, are then required for the refolding of the SNARE SNAP-25, converting it into an active form [19]. Chaperones can also disassemble native complexes such as clathrin cages [20] and they may target short-lived or stress-damaged proteins to proteasomal or lysosomal degradation and reorient mutant proteins prone to aggregation back on track of the native pathway, to undergo functional folding and assembly [4].

The expression of molecular chaperones is markedly increased under different environmental stress conditions, for example following hyperthermia or heat shock, hypoxia, oxidative stress, or exposure to toxins [5, 21–23]. The stress response is thought to be activated by the accumulation of unfolded or misfolded proteins, eliciting chaperone expression by turning on a signaling pathway that engages the transcription factor heat shock factor 1 (HSF-1) [22–25]. Under stress, such as heat shock, all organisms massively synthesize heat-shock proteins (HSPs), many, but not all, belonging to the molecular chaperone category. Compared to average human genes, members of the human “chaperome” network [26] are 20 times more likely to be stress-inducible [21]. Yet, noticeably, two-thirds of the human chaperome is constitutively expressed without stress and constitutes up to 10 % of the total protein mass of HeLa cells [27]. In young animals, molecular chaperones can effectively retard the formation of cytotoxic protein aggregates such as fibrils, tangles, and amyloids, which are hallmarks of degenerative diseases, such as Alzheimer’s, Parkinson’s, Huntington’s, diabetes type 2, and Prion diseases.

The involvement of molecular chaperones in neurodegenerative diseases can be exemplified with the particular case of Parkinson’s disease (PD). Indeed, Hsp90, Hsp70, Hsp60, Hsp40, and Hsp27 were found in Lewy bodies and Hsp70 in particular was inferred to be an important chaperone to mitigate α-synuclein toxicity [28–31]. Further, exposure of cells and whole mice to toxins like rotenone or 1-methyl-4-phenyl-1,2,3,6-tetrahydropyridine, or to the proteasome inhibitor lactacystin, showed a marked increase in chaperone levels, particularly of Hsp70 [32, 33]. Likewise, targeted overexpression of α-synuclein using viral-vector in the *substantia nigra* of mice resulted in increased mRNA levels of Hsp70, Hsp40 and Hsp27 [34]. Moreover, the sequestration of molecular chaperones into protein aggregates results in their cellular depletion and thus a subsequent loss of chaperone function that may promote neurodegeneration [35]. Consistently, in vitro, α-synuclein oligomers caused the depletion of Hsp40 (DnaJ) rendering the Hsp70 machinery (DnaK–DnaJ–GrpE) inefficient at unfolding/refolding misfolded proteins [36]. A systematic study of the interaction of several small Hsps (αB-crystallin, Hsp27, Hsp20, HspB8, and HspB2B3) showed that transient binding to the various forms of α-synuclein resulted in the inhibition of mature α-synuclein fibril formation [37]. Further, in vitro experiments showed that the small HSP, αB-crystallin (HspB5) can mediate the depolymerization of α-synuclein fibers with the help of other chaperones, including Hsp70 and its co-chaperones [38]. Moreover, in an in vitro system, mammalian Hsp110 can synergize Hsp70 to drive the catalytic disaggregation of α-synuclein amyloid fibrils [39]. All these studies show a close linkage between cellular stress, toxic protein misfolding, and chaperone induction, suggesting that protein misfolding diseases could result from chaperone failure and that the artificial increase of the cellular chaperone load by ectopic expression or drugs mimicking various stresses could combat protein misfolding diseases [4].

Noticeably, under mildly stressful conditions, protein aggregates in the cell and in vitro can serve as nucleating seeds to the aggregation of other metastable proteins that would otherwise spontaneously revert to the native state [40, 41]. Chaperones are thus key factors to neutralize the aggregation seeds, thereby disallowing a prion-like propagation-of-aggregation effect even among regular labile proteins [4, 40]. Hence, small amounts of arsenite-, lead-, or cadmium-induced protein aggregates can serve as seeds that commit other labile proteins in excess to misfold and aggregate even after all traces of heavy metals have been removed from the seeds. Fortunately, this seeding process can be effectively counteracted by “holding” and unfolding chaperones such as Hsp70 and CCTs [42, 43].

## The various chaperone activities

Whereas many but not all chaperones can passively bind misfolding proteins and thus arrest further aggregation into insoluble, potentially cytotoxic species, chaperone activity goes much beyond mere passive stoichiometric binding of metastable polypeptide species. Because binding or tight holding are not catalytic processes, the term “holdase” that is often used in the chaperone literature should be avoided. Moreover, many molecular chaperones function under physiological conditions as regulators of native protein folding, translocation, and assembly that do not call for their ability to prevent aggregation. Significantly, at least three out of five main chaperone families can act as bona fide polypeptide unfoldase enzymes.

In unstressed cells, molecular chaperones play a central role in protein homeostasis and regulate structural transitions between native and “alternative” states of proteins, such as between the oligomeric active versus the monomeric inactive states of native IκB, caspases or HSF-1 [44–47], or between inactive and active steroid hormone receptors [48–50]. In stressed cells, molecular chaperones become a primary line of cellular defenses against stress-induced protein misfolding and aggregation events [51] that otherwise become increasingly toxic by compromising the stability of other proteins and the integrity of membranes [52]. In aging mammalian neurons, toxic protein aggregates generally cause neuro-inflammation, oxidative stress, apoptosis, and tissue loss, leading to neurodegeneration and diseases.

Most molecular chaperones fall into five main families of highly conserved proteins: the Hsp100s (ClpB), the Hsp90s (HtpG), the Hsp70/Hsp110 (DnaK), Hsp60/CCTs (GroEL), and the α-crystalline-containing domain generally called the “small Hsps” (IbpA/B) (*Escherichia coli* orthologues shown in parentheses). Apparently, all families share the ability to screen for proteins with hydrophobic residues that are abnormally exposed to the solvent, and are thus prone to associate and form stable inactive aggregates [3, 4, 53]. With the exception of the small Hsps, the major classes of molecular chaperones are also ATPases, suggesting that their function can implicate an ATP-driven increase of the free energy in their bound misfolded or alternatively folded polypeptide substrates [54].

## Chaperones with holdase activity

The first in vitro chaperone assay showed that the *E. coli* Hsp60, GroEL, could passively prevent the aggregation of a urea-, acid- or Guanidium HCl-denatured RuBisCO substrate. Importantly, in addition to the GroEL ability to “hold” the inactive RuBisCO in a soluble inactive state, the addition of GroES and ATP subsequently released the substrate from the holding GroEL, which then refolded into native active RuBisCO [55]. Yet, rather than referring to this remarkable ability of chaperones to drive the stringent native refolding of unfolded proteins, which would have otherwise remained inactive and aggregated, most subsequent papers chose to adopt the definition of chaperone activity as being the ability to prevent aggregation of heat- or Guanidium HCl-denatured proteins. In addition to chaperonin, many but not all chaperone families, including Hsp40, Hsp90, CCTs, and sHsps but not Hsp104 (ClpB), were shown to effectively prevent the aggregation of proteins in the absence of ATP [5, 56]. The term “holdase” thus was dubbed to describe the physical tight interaction of a chaperone with a non-native unfolded or misfolded polypeptide, which thus became prevented from forming larger aggregates that scatter light [4]. The “holdase” activity could be qualitatively observed with a previously unfolded polypeptide set to aggregate in a fluorometer cuvette: the presence of a given amount of chaperone caused the lowering and slowing down of the time-dependent increase in the light scattering signal [57]. However, the “holdase” activity of chaperones remained mostly a mere qualitative observation, since light-scattering assays suffer from low sensitivity and signals lack a direct connection with the size distribution of the aggregates.

**Fig. 1:**
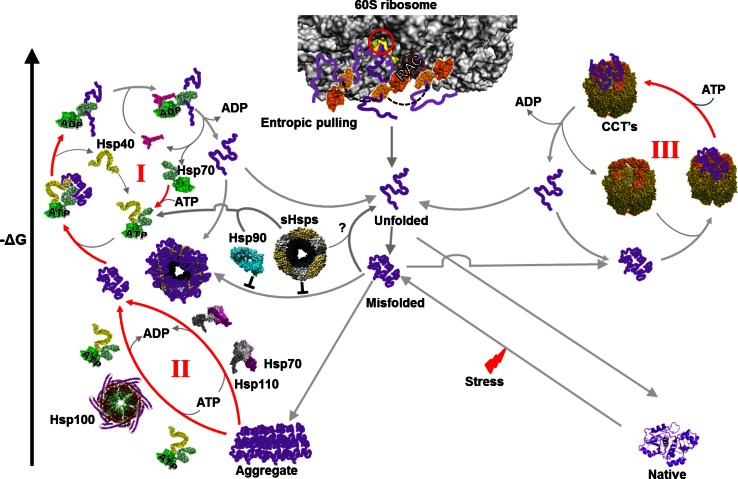
Folding of nascent and misfolded polypeptides by the cytosolic chaperone network. A newly synthesized polypeptide emerging from the eukaryotic ribosome (PDB:3O2Z) tunnel (*red circle*) encounters in a typical eukaryotic cell such as yeast, ribosome-associated chaperones that can apply an entropic pulling force to unfold misfolded secondary structures in the growing nascent polypeptide chain. Upon exposure of the nascent polypeptide to the crowded environment of cytosol it may expose hydrophobic residues leading to misfolding. The misfolded conformers may then become a substrate of Hsp70 (PDB:1KHO) system (Hsp70–Hsp40 and nucleotide exchange factor, PDB:1DKG), which by reiterative cycles of binding, ATP-fueled unfolding and spontaneous refolding, converts the misfolded polypeptide into a native protein (cycle I). In case of failure, the misfolded substrate can bind instead to holding chaperones such as Hsp90 or sHsps that may keep the substrate in a non-aggregated, folding competent state, which may be subsequently passed on to the unfolding machinery of Hsp70 system for refolding to the native state. Possible unfolding of misfolded substrate by sHsps, structure adapted from [157], is unclear and is shown as a *question mark*. The aggregated protein in the cytosol of metazoans can be reverted to the native state by the Hsp110–Hsp70 system (PDB:3C7N) and also by the Hsp100s (structure adapted from [158]) and Hsp70 system in yeasts and plants (cycle II). In case of failure, the misfolded polypeptide can bind instead the CCT chaperonin (PDB:4A13), where it will undergo cycles of binding, unfolding, and ATP-fueled release, leading to the native state (cycle III). The structures are from highly homologous chaperone orthologs from various organisms, because they are not all available from yeast

A further depreciation of the concept that chaperones are “holdases”, is the fact that the Hsp100/ClpB chaperones are unable to passively prevent the aggregation of unfolding or unfolded polypeptides, but rather act as very effective disaggregase chaperones, which together with HSP70, use ATP to forcefully solubilize already preformed, stable protein aggregates [58]. Although not all chaperones have a “holdase” activity, there is a general agreement to describe the activity, at least of the small-HSPs, as such, possibly because the α-crystallin domain-containing small heat shock protein (sHSPs) are devoid of ATPase activity of their own. Thus, under stress conditions, small-Hsps like Hsp25 or IbpB can bind very tightly to non-native unfolding or unfolded proteins and maintain them in an inactive non-aggregated state, which may be subsequently fed to an ATP-dependent unfoldase chaperone machinery such as Hsp70–Hsp40, to become reactivated after the stress (Fig. 1) [59–61]. Several in vitro studies using purified sHsps from various organisms have demonstrated that sHsps can effectively prevent the thermal aggregation of other proteins in an ATP-independent manner. They describe “holding” by sHsps as a single step, which is nearly irreversible in biological timescales, rather than a dynamic binding/release process [62–65]. Whereas small Hsps (sHsps) do not generally drive dissociation at a useful rate and are energy independent, other chaperones can bind (and unfold) already-formed stable misfolded proteins, as in the case GroEL and CCT, which need energy to drive dissociation at a useful rate. Yet, other chaperones bind and disaggregate already-formed large insoluble stable protein aggregates, as in the case of Hsp110–Hsp70 and Hsp100–Hsp70 bichaperone machineries [58, 66]. It should be noticed that because all enzymes need to bind their substrate, it is futile to mention protein binding as a particular property of the chaperones. Even when non-enzymatic polypeptides bind other macromolecules, as with histones binding DNA, this does not qualify them to be named DNA *bindases* or *holdases*. In another example, glucose-6-phosphate dehydrogenase (G6PDH) binds glucose-6-phosphate (G6P) and NADP with high affinity and converts them into 6-phosphoglucono-lactone and NADPH + H [67]. It would be misleading and poorly informative to name G6PDH “G6P holdase”. Thus, it would seem inappropriate to assign a holdase activity to chaperones that drive the forceful unfolding and translocation of polypeptides across membranes [20]. Polypeptide *translocases*, *unfoldases*, and/or even *pullases* would better fit the definition of their function.

In order to label a given protein as an enzyme, it should carry basic properties common to all enzymes. Like all catalysts, it should act by way of lowering the energy of activation of a spontaneous reaction and thus increase the rate at which equilibrium is reached and it should not remain stably associated to its products. It should not be consumed by the reactions, nor should it alter the equilibrium of the catalyzed reaction. The International Union of Biochemistry and Molecular Biology (IUBMB) has formulated several principles to name new enzymes. First, the name should end with suffix “-ase”, implying that it has a catalytic mode of action, driving iterative cycles of substrate binding, substrate conversion into product, and product release. The use of the suffix “-ase” is strongly discouraged for non-enzyme molecules. Second, efforts should be made to classify new enzymes among the six existing classes of the *Oxidoreductases*, *Transferases*, *Hydrolases*, *Lyases*, *Isomerases*, and *Ligases*. Third, enzymes should be named according to the main reaction they catalyze (Enzyme Nomenclature 1992, Academic Press, San Diego, California, ISBN 0-12-227164-5).

The term “*holdase*” is thus an oxymoron: Either a chaperone is an enzyme deserving the suffix “-ase”, in which case it should act as a catalyst, i.e., it should also be able to carry many cycles and in particular to *release* its products within a biologically relevant time-scale at the end of every cycle, or it should not harbor the suffix “-ase” and rather be called “holding” chaperones. Noticeably, even the small HSPs for the activity of which, the term “*holdases*” is most often used, can also accelerate the native refolding of artificially unfolded proteins, raising the possibility that small HSPs might also act as polypeptide foldases [68].

## Chaperones with catalytic polypeptide unfoldase activity

**Fig. 2:**
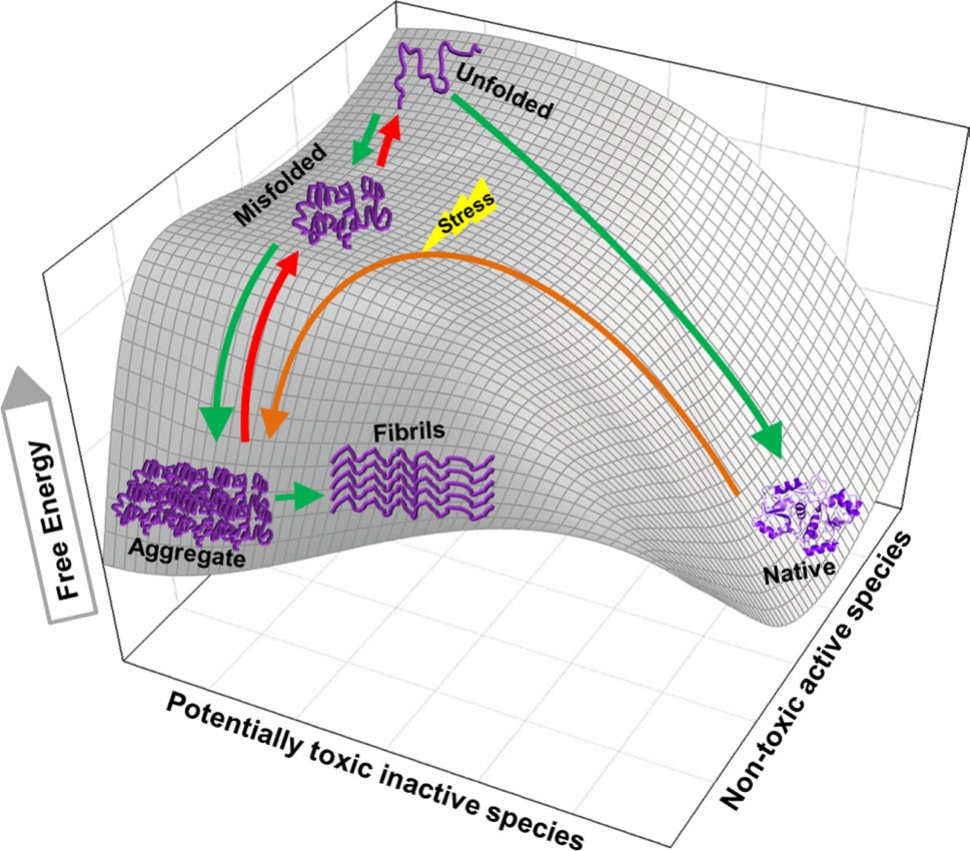
Disaggregating and unfolding chaperones transiently increase the free energy of misfolded or alternatively folded substrates but not of natively folded products. The 3D mesh-plot shows a typical unfolded polypeptide with the highest free energy, which can spontaneously reach to lower free energy states, either by folding to the native conformation (*right*) or by misfolding to the aggregated state (*left*). When conditions are not favorable for native folding, the unfolded polypeptide may prefer undertaking the misfolding pathway to aggregation (*green arrows*). Native proteins under stress gaining free energy may partially unfold to a state from which it can readily seek a more stable misfolded state [3] and further aggregate (*brown arrow*) [2]. ATP-fueled unfoldase chaperones drive the substrate uphill the free energy barrier (*red arrows*) by converting stable aggregates and misfolded species into unfolded products with a higher free energy. From there, if conditions are favorable to the end product, the unfolded species can collapse to the stable native state

Molecular chaperones such as Hsp70, Hsp110, Hsp100, or Hsp60s can use ATP to unfold stable misfolded or aggregated proteins and convert them into natively refoldable species [6, 13, 66]. Hsp70, in collaboration with co-chaperones Hsp40 and nucleotide exchange factor (NEF), function as an efficient unfolding or disaggregation machinery [69–71]. The bacterial Hsp70 system that includes DnaK (Hsp70), DnaJ (Hsp40), and GrpE (NEF), can work at *V*_max_ in multiple turnovers, converting a molar excess of stable inactive misfolded protein species into the active native state, in a strict ATP-dependent manner [13]. This conversion by Hsp70s of stable misfolded polypeptides into the native species is accomplished by working against a free energy barrier, converting a stable misfolded protein with a low free energy into a transiently unfolded species with a higher free energy, which, after release may spontaneously fold to a native species with a lower free energy (Fig. 2). Demonstrating that unfolding of stable misfolded species is highly conserved in evolution, a similar unfolding mechanism leading to spontaneous native refolding was shown in the case of human Hsp70 (and Hsp40) and its ortholog Hsp110 (with Hsp40), which was indistinguishable from that of bacterial DnaK, except that is was energetically much more expensive [66] and also that NEF activity was not involved, as this was the case in the bacterial system [13]. Likewise, a similar unfolding mechanism leading to spontaneous native refolding was shown in the case of bovine CCT, which was indistinguishable from that of bacterial GroEL [12]. Indeed, the mere binding of a stable misfolded fluorescently labeled rhodanese was shown by fluorescent spectroscopy to cause significant unfolding in the substrate, which was exacerbated further upon ATP addition [6].

The catalytic unfolding of stable misfolded polypeptides by Hsp70s and Hsp60s, like other enzymatic reactions, starts with the misfolded polypeptide acting as a high-affinity substrate, the unfolded species as an unstable intermediate of the reaction with a yet higher affinity for the catalytic site of the enzyme, and the natively refolding/refolded protein as the low-affinity product of the reaction. Owing to their demonstrated abilities to convert a stoichiometric excess of misfolded polypeptide substrates through successive cycles of binding, unfolding, release, and native refolding, Hsp70/Hsp110s and Hsp60/CCTs have thus earned the qualification of bona fide polypeptide unfoldases [13, 66].

The mechanism by which Hsp70 can use ATP hydrolysis to pull and unfold polypeptide segments in stably misfolded and aggregated proteins, or pull and unfold alternative proteins across the membranes, involves a possible direct unfolding effect on the bulky substrate, possibly by the clamping of the lid of the chaperone protein binding domain towards its base [72, 73], as well as by a subsequent more global cooperative entropic pulling action [74] between the Hsp70 molecule and the import pore or between several Hsp70s concomitantly bound at different places on the same misfolded polypeptide [20]. In vitro, the ATPase chaperonins GroEL (organized in two homo-heptameric rings) and CCT (organized in two hetero-octameric rings) that consequently show multiple substrate binding sites, can mediate several consecutive cycles of binding-unfolding-release-refolding [12, 75, 76]. ATP is mostly used to fuel the forceful eviction of high-affinity over-sticky intermediates that following several cycles may start to accumulate on catalytic unfoldase sites and act as competitive inhibitors of the catalytic unfoldase reaction [12]. Other chaperones, such as the small HSPs, which are not ATPases, could also act as polypeptide unfoldases, provided an eviction mechanism of the over-sticky intermediates exists. In the case of the small HSPs, the Hsp70 chaperone system has been shown to carry such a regenerative function for over-sticky misfolded protein substrates associated to sHSPs [61]. Recently, bacterial and mammalian Hsp90 has been shown to carry an ATP-dependent activity leading, in collaboration with the Hsp70 chaperone system, to the refolding of some misfolded polypeptides such as luciferase and of native proteins, such as p53 (Fig. 1) [77–79].

Noticeably, depending on the conditions, catalytic unfoldases can reversibly switch into “holding” chaperones. Under heat stress, heat-labile proteins such as firefly luciferase or malate dehydrogenase tend to spontaneously convert into stable inactive aggregates [80, 81]. Under such unfavorable unstable conditions for the native product of the reaction, an active polypeptide unfoldase in the cell could result in the deleterious acceleration of protein misfolding and aggregation [80]. Thus, whereas at 25 °C, equimolar GroEL and GroES can optimally catalyze the in vitro release and the native refolding of a pre-bound inactive malate dehydrogenase, at 43 °C, the affinity of GroES for GroEL is reversely decreased with the consequence that as long as the denaturing elevated temperature is maintained, GroEL binds (but does not release) the misfolded MDH substrate, despite the presence of equimolar GroES and ATP in the solution. Thus, the GroEL-bound MDH is prevented from aggregating until the temperature is decreased and GroES rebinding can resume, inducing substrate release and native refolding [82]. Similarly, the chaperone activity of bacterial DnaK–DnaJ–GrpE can be reversibly arrested at elevated temperatures due to the reversible decrease of the substrate-release factor GrpE at high temperature [82, 83].

Given that various molecular chaperones are expected to arrest their catalytic polypeptide unfoldase activity under stress and thus become transiently passive “holding” chaperone that merely prevent aggregation, they may need to be present at much higher cellular concentrations than if they were only catalytic unfoldases. This could account for the observed very high cellular concentrations of core members of the chaperome network, which can reach up to 10 % of the total to protein mass [27]. Noticeably, an abundant chaperone is not evidence of its inefficiency as an unfolding catalyst. Suffice it to be slow, as in the case of RuBisCO, which owing to its relative slowness at catalytically incorporating inorganic carbon into the planet’s food chain [84], it also needs to be the most abundant protein in the biosphere [85].

## Chaperones with disaggregase activity

Bacterial Hsp70 (DnaK), in the presence of its DnaJ and GrpE co-chaperones and ATP, has been shown in vitro to be able to convert stable preformed small soluble aggregates [70]. Yet, for the disaggregation-unfolding-refolding reaction to be optimal, a large molar excess of the Hsp70 chaperone over the substrate was necessary, a constraint that could be explained by a mechanism of entropic pulling [69, 70, 74]. To alleviate the necessity for a non-physiological excess of Hsp70 over its aggregated substrates, nature may have designed specific Hsp70 co-chaperones in the form of the AAA+ rings of ClpB/Hsp104 or the Hsp70-like Sse/Hsp110s.

Hsp100s (also named ClpB in bacteria, Hsp104 in yeast, and Hsp101 in plants) are AAA+ hexameric ring-like chaperones termed “*disaggregases*” because they act as nanomachines harnessing the energy of ATP hydrolysis to the forceful unfolding and solubilization of large stable protein aggregates to be converted in collaboration with Hsp70s (DnaK) into natively refolded proteins (cycle II, Fig. 1) [58, 86]. The disaggregase activity of the bacterial Hsp100–Hsp70 (ClpB-DnaK) system is attributed to both their individual and reciprocally regulated concerted unfolding actions on the stable misfolded and aggregated substrates (Fig. 1) [58, 70, 87, 88]. Demonstrating that disaggregation of stable misfolded species is highly conserved in evolution, a similar disaggregation mechanism, leading to spontaneous native refolding, was initially shown in the case of yeast Hsp70 (and Hsp40) and the ClpB ortholog Hsp104 [89, 90]. Unlike yeast and plants, metazoans lack bona fide ClpB/Hsp104-like disaggregases. Yet, they possess another disaggregating chaperone couple composed of a bona fide Hsp70, loosely associated to an evolutionarily related chaperone called Hsp110. Noticeably, Hsp110 in animals, also called Sse in yeast, structurally and functionally belongs to the Hsp70 family [66, 91]. It is not to be confused with the Hsp100 chaperones, which are unrelated to Hsp70s and are rather AAA+ proteins. Hsp110 and Sse were initially described as mere NEF of the Hsp70 (Ssa in yeast) chaperones [92–94] and indeed, even without ATP, human Hsp110 was shown to induce the release of an unfolded substrate from human Hsp70, exactly as bacterial GrpE induced the release of unfolded protein bound to bacterial DnaK [13, 66]. Moreover, suggesting a tight link between ATP-fueled unfolding and disaggregation, Hsp110 with Hsp40 (but without Hsp70) was found to be able to unfold misfolded luciferase monomers, but not large aggregates [66]. In contrast, human Hsp110 and Hsp70 chaperones (with Hsp40) were shown to concertedly act as equal partners that use ATP hydrolysis to disaggregate and unfold large stable luciferase aggregates [39, 66, 91, 95, 96] (Fig. 1). The NEF, Bag1, could not substitute for Hsp110 as a co-chaperone of the disaggregation mechanism [95]. Interestingly, the cytoplasm of plants, yeast, and fungi harbors both Hsp100–Hsp70 and Hsp110–Hsp70 disaggregating machineries, suggesting that the two do not quite overlap in terms of their respective aggregate specificity. Thus, although the human cytoplasm propitiously carries at least one effective disaggregation system (Hsp110–Hsp70), compared to yeast it may still suffer from lacking the Hsp104–Hsp70-based disaggregating machinery, a loss of function that possibly contributes to the excessive sensitivity of aging metazoan neurons to toxic protein aggregates.

## Chaperones with polypeptide translocase activity

Trigger factor (TF), initially described as a putative peptidyl-prolyl *cis*–*trans* isomerase, is a bacterial chaperone that transiently associates to the ribosomal protein L23, where the growing polypeptide chain exits the ribosome and enters into the crowded cytosol. TF lacks ATPase activity. It passively interacts with most polypeptides early during synthesis where it possibly acts as a peptidyl prolyl isomerase, i.e., as a *foldase*, accelerating native folding. It is the first chaperone to associate with nascent chains, thereby acting upstream to the cytosolic DnaK and GroEL *unfoldase* chaperone machineries [97–103]. Already during passage through the ribosomal tunnel, nascent chains may acquire some wrong secondary structures [104]. TF is reported to “hold” the nascent chain and thus apply some pulling and unfolding on the exiting polypeptides [105, 106]. Moreover, TF’s particular shape allows it to mould the de novo folding of small polypeptide domains [107] in a direct assisted-folding mechanism of the nascent chains that is clearly distinct from the pulling and unfolding mechanism by DnaK. In addition to its role at the ribosomal exit in de novo protein folding, in vitro assays with artificially unfolded polypeptides have shown that TF can also promote proper native refolding without ribosomes, by transient holding/pulling and/or by acting as a peptidyl prolyl* cis*-*trans* isomerase [108–110].

Eukaryotes have evolved a different co-translational folding machinery that involves specific variants of Hsp70 and Hsp40. In yeast, this system consists of an Hsp70-like chaperone, *Ssz1* (Hsp70L1 in humans), a J-protein, *Zuo1* (MPP11 in humans), and a stable, ribosome-associated heterodimer named RAC, with two functionally interchangeable Hsp70s, *Ssb1* and *Ssb2*, thus forming a chaperone triad at the ribosome tunnel exit (Fig. 1). When expressed in *S. cerevisiae*, *E. coli* TF can bind to the yeast ribosomes and partially complement a knockout of the yeast ribosomal chaperone triad [111]. In mammals, *Ssb* is absent, but is functionally replaced by the abundant cytosolic Hsc70 [112, 113]. RAC acts as a co-chaperone that stimulates the ATPase activity of *Ssb* (Hsp70) through the J-domain of Zuo1 [114]. Like other J-proteins, Zuo1 associates with ribosomes and target Hsp70 (Ssb) onto the growing polypeptide at the exit of the ribosomal tunnel [113] (Fig. 1).

Moreover, Hsp70 chaperones can assist in general in the post-translational translocation of polypeptides across the membranes of organelles, such as the *endoplasmic reticulum* (ER), mitochondria, and chloroplast [115–117]. The energy necessary to unfold a cytoplasmic precursor protein and translocate it unidirectionally into an organelle through a narrow pore allowing only unfolded polypeptides to cross, may come from the membrane potential driving the polypeptide initial insertion into the pore [118] and ATP hydrolysis by the Hsp70 (mtHsp70, also known as Mortalin, in mitochondria, and BiP in the ER) acting as an import motor on the acceptor side of the membrane [119–122]. The reversible docking of mtHsp70 to the pore and binding (locking) onto the entering polypeptide is simultaneously regulated by the pore-anchoring proteins Tim44 and the J-domain proteins Pam18/Pam16, and by the nucleotide exchange factor, Mge1. Similarly, the post-translational translocation of specific proteins across the membrane of the ER to the lumen involves a pore-like protein, Sec63, which exposes a J-domain on the lumen side and by doing so, acts as a reversible anchor to BiP, the ER Hsp70 [116].

Two divergent models were initially proposed to explain the mechanism of chaperone-mediated unfolding and unidirectional translocation of precursor protein to the mitochondria. The first was the *Brownian ratchet* model, where polypeptide-bound mtHsp70 was suggested to act as a ratchet that passively prevents backsliding to the cytoplasm and thus driving the polypeptide’s inward translocation [121, 123–126]. The second model, called *power stroke*, suggested that a polypeptide and pore-bound mtHsp70 could use the energy of ATP hydrolysis to undergo a conformational transition that, exploiting the pore as a *fulcrum*, would act as a lever arm applying an inward force on the polypeptide and causing its unfolding on the cytoplasmic side and subsequent import [127–133]. Taking advantage of new mechanistic information in the absence of an import pore, on the involvement of Hsp70 (DnaK) and Hsp40 (DnaJ), in the ATP-fueled solubilization and unfolding of stable protein aggregates, a unifying model called *entropic pulling* was proposed. It reproduced the combined effects of the two models above by drawing attention to the fact that upon release from the pore (or from the aggregates), an Hsp70 molecule locked onto a substrate polypeptide applies a pulling force of entropic origin on the polypeptide that needs neither coordinated structural transformations in Hsp70 nor a mechanical fulcrum. Instead, the pore and the surrounding membrane, or a large aggregate, constrain the freedom of movement (thus, the entropy) of the polypeptide–chaperone complex, with an effect that decreases as the distance of the complex from the constraint increases. As a consequence, because of thermodynamics, the complex is entropically pulled away from the pore or from the aggregate. In entropic pulling, the energy of ATP is not directly converted into a mechanical force, as postulated by the power-stroke model, but rather into an indirect thermodynamic force. In the case of translocation, such force remains operative only until 30–40 amino acids have been imported, reducing thereafter to a pure ratchet, unless a new Hsp70 molecule binds [74, 134].

## Chaperones with targetase activity

J-domain proteins are also described as holdases [135] but they are principally obligate co-chaperones of the Hsp70/Hsp110s ATPases. J-proteins bind first to misfolded [69, 70], alternatively folded chaperone substrates [136], or to unfolded polypeptides at the ribosomal exit pore [137], or at the import pores of mitochondria or ER [138–140], and may thus attract Hsp70 molecules onto their putative protein substrates. The docking of the highly conserved J-domain to the nucleotide binding domain of Hsp70 (or Hsp110) molecule poises the latter to hydrolyze ATP and, by allostery, causes the locking of the protein binding domain upon a misfolded, unfolded, or alternatively folded polypeptide substrate. The locking of a single Hsp70 molecule may cause the global unfolding of a single domain protein, as in the case of firefly luciferase that acts virtually as a single domain protein [13]. However, in the case of a multidomain polypeptide, such as G6PDH, the locking of a single Hsp70 is expected to cause only a local partial unfolding of the bound polypeptide segment [69, 141, 142]. In this case, the collaborative action of several concomitantly bound Hsp70s at different places on the same polypeptide can cause an additional pulling effect of entropic origin, leading to the global unfolding of the protein (cycle I and II, Fig. 1) [69, 74]. Subsequent to Hsp70-mediated unfolding of the substrate, a nucleotide exchange factor, such as bacterial GrpE, or eukaryotic Bag3, may cause the dissociation of ADP and of the unfolded product from Hsp70 [143]. The product may then spontaneously refold to the native state [13]. If at this stage, misfolding happens rather than native refolding, further unfolding cycles may be needed until all molecules have reached the most stable native state (cycle I, Fig. 1).

Recently, it was reported that mere binding of Hsp40s (DnaJ) could cause some unfolding within a polypeptide [144]. This is, however, not a general effect as a large molar excess of bacterial DnaJ was shown not to disturb wrong beta sheets in a stable misfolded luciferase species, whereas substoichiometric amounts of DnaJ supplemented with DnaK and ATP readily unfolded it [13]. In the cytoplasm and the ER of human cells, the total copy number of J-proteins is respectively 6.4- and 9.6-fold less than the sum of the copy number of Hsp70 and Hsp110 present in the same compartments, confirming that J-proteins unlikely act as equal stoichiometric partners of the Hsp70/Hsp110 unfoldase machinery, but rather as catalysts [27]. Indeed, in vitro refolding assays show that J-proteins (Hsp40s) are optimally acting when present in sub-stoichiometric ratios compared to their Hsp70 partners as in the cell [36, 91]. Thus, 20 times less DnaJ than DnaK can drive at half optimal rates the active refolding of stably heat-preaggregated G6PDH enzyme [36]. This apparent catalytic mode of action by J-proteins implies that J-proteins should not act as holding chaperones but would rather need to be able to readily dissociate from their substrates as soon as Hsp70 has hydrolyzed ATP and thus evicted the bound J-domain from the nucleotide binding domain, while concomitantly locking and unfolding the polypeptide substrate in the protein binding domain. Remarkably, once the DnaJ has bound to an aggregated substrate and recruited the DnaK and once ATP-fueled DnaK locking onto the misfolded polypeptide has caused the substrate to unfold, this disentanglement is observed to effectively drive DnaJ dissociation, likely because the ATP-fueled DnaK-mediated unfolding destroyed the high affinity DnaJ-binding sites (cycle I, Fig. 1) [36, 145]. In the cell, this may be illustrated in the case of the J-protein auxilin, which in collaboration with Hsc70 mediates the de-oligomerization of clathrin baskets in an ATP-dependent manner. Auxilin, which is ~2,700 times less abundant than Hsc70 in the cytoplasm, initially binds to the clathrin heavy chain, then it entraps Hsc70 by way of inserting its high affinity J-domain in the nucleotide-binding domain. This triggers ATP hydrolysis and causes the locking of the protein binding domain of Hsc70 onto the heavy chain [27, 146, 147]. In vitro, the binding of auxilin to clathrin saturates at three auxilin molecules per clathrin triskelion [148]. However, when auxilin acts as co-chaperone for the targeting Hsc70 onto clathrin baskets in the uncoating reaction, only catalytic amounts of auxilin are required, compared to the Hsc70 and the triskelions [149]. Thus, rather than being referred to as *holdases*, J-proteins in general would better answer to the definition of the “Hsp70/110-*targetases*” (Table 1).

**Table 1:** The major conserved families of molecular chaperones with their established functions, as well as their yet-to-be-demonstrated possible additional functions

Function(s)	Hsp100 (ClpB)	Hsp70/110 (DnaK)	Hsp60 (GroEL)	Hsp90 (HtpG)	Small-Hsps	J-Proteins
Generally accepted function	Disaggregase [58, 86]	Unfoldase [13] Translocase [159]	Holding [160] Folding [161]	Holding [162]	Holding [61]	Holding [135]
Possible additional function	Unfoldase [163]	Disaggregase [39, 66, 70] Holding [164]	Unfoldase [6, 12]	Unfolding [77]	Folding [68]	Hsp70/110 Targetase [74]

In conclusion, various well-known conserved families of molecular chaperones share the ability to bind more or less tightly and less or more reversibly, to misfolded, aggregated, unfolded, or alternatively folded proteins, but not to native proteins. They may, however, strongly differ in the outcome of polypeptide binding. Upon binding, some chaperones may cause spontaneous unfolding of the polypeptide substrate, others merely prevent aggregation, and yet others may need to use the energy of ATP hydrolysis to forcefully unfold, pull apart, de-oligomerize, and/or disaggregate various polypeptide substrates. Because passive, tight polypeptide binding by a chaperone is not a catalytic process, the suffix “-ase” should be avoided, and the term “holding” chaperones used instead. Because there is a growing number of molecular chaperones that upon substrate binding and unfolding, end up releasing their bound polypeptides in a folding competent state within a biologically reasonable time scale, these chaperones deserve the label of polypeptide *unfoldases* acting as bona fide enzymes. They are functionally related to the class 5 isomerases. Like peptidyl prolyl *cis–trans* isomerases, Hsp70s, or GroEL/CCTs, they do not obligatorily require the breakage of a covalent bond in their polypeptide substrates to catalytically unfold them. Like topoisomerase, they may need to hydrolyze ATP to drive the conformational changes in the misfolded substrates, although catalysis does not change the overall chemical composition [54].

Type I and type II J-proteins can apparently bind to unfolded or misfolded polypeptides and thus incidentally also prevent their aggregation to some degree. Other J-domain proteins can bind to alternatively folded substrates, such as sigma 32 [150], and SNARES that are substrates that do not tend to aggregate under physiological conditions. Likewise, auxilin is a J-protein that can bind only to the alternatively folded clathrin cages and Pam16/18 of the mitochondrial import pore do not directly bind to any substrate protein per se but only indirectly by way of the nearby pore [18, 20, 151, 152]. Rather, J-domain proteins principally act as chaperone targeting devices. In sub-stoichiometric amounts, they drive the binding and “locking” of Hsp70s and/or Hsp110s onto their various alternative, misfolded, or translocating unfolded polypeptides substrates, leading to effective pulling, unfolding, and, upon product release, to native refolding. J-domain co-chaperones should thus best be termed *Hsp70/110* *targetases*.

Table 1 summarizes the main classes of conserved molecular chaperones, the various well-established and yet ill-characterized molecular activities, from passive holding and targeting to spontaneous and ATP-fueled catalytic unfolding, disaggregating, pulling, and translocating.

Proteins that need to alternate between various states in order to carry their physiological functions may also bear an intrinsic sensitivity to environmental changes. Thus, labile proteins in cells under mild stresses may tend to unfold and transiently expose hydrophobic residues to the crowded hydrophilic environment, which, depending on the stress intensity and duration, may lead to intra-molecular misfolding and the gradual formation of increasingly stable inter-molecular ensembles called aggregates or amyloids. Likely owing to wrong hydrophobic interactions, the earliest forms of misfolded and aggregated species can compromise the integrity of cellular membranes and the stability of other labile native proteins [153–155]. Moreover, in animal cells, misfolded conformers induce apoptotic signals, which can lead to a gradual loss of neural tissue, as in Alzheimer’s and Parkinson’s diseases [4]. These ensuing degenerative conditions are late-onset diseases, likely because they correlate with an age-dependant decreased ability of neurons to sense various abiotic stresses and thus to appropriately produce protective HSPs [156]. In youth, however, when the cellular stress response is optimal, the HSP chaperone network can effectively prevent and avert the formation of early misfolded and aggregated proteotoxic conformers. It is therefore essential to understand which of the specific holding, unfolding, targeting, pulling, and/or disaggregating mechanisms the various members of the cellular chaperone network are using, individually and in collaboration, to reduce proteotoxic species and convert them into harmless, degraded or “rehabilitated” functional native proteins [100]. Detailed knowledge of the various chaperone mechanisms is central to the design of future chaperone-based therapies against protein conformational diseases and aging.
